# Random subwindows and extremely randomized trees for image classification in cell biology

**DOI:** 10.1186/1471-2121-8-S1-S2

**Published:** 2007-07-10

**Authors:** Raphaël Marée, Pierre Geurts, Louis Wehenkel

**Affiliations:** 1 GIGA Bioinformatics Platform, University of Liege, B34 Avenue de l'Hopital 1, Liege, 4000, Belgium; 2 Bioinformatics and Modeling, Department of Electrical Engineering and Computer Science & GIGA Research, University of Liege, B28 Grande Traverse 10, Liege, 4000, Belgium

## Abstract

**Background:**

With the improvements in biosensors and high-throughput image acquisition technologies, life science laboratories are able to perform an increasing number of experiments that involve the generation of a large amount of images at different imaging modalities/scales. It stresses the need for computer vision methods that automate image classification tasks.

**Results:**

We illustrate the potential of our image classification method in cell biology by evaluating it on four datasets of images related to protein distributions or subcellular localizations, and red-blood cell shapes. Accuracy results are quite good without any specific pre-processing neither domain knowledge incorporation. The method is implemented in Java and available upon request for evaluation and research purpose.

**Conclusion:**

Our method is directly applicable to any image classification problems. We foresee the use of this automatic approach as a baseline method and first try on various biological image classification problems.

## Background

With the improvements in biosensors and high-throughput image acquisition technologies, life science laboratories are able to perform an increasing number of experiments that involve the generation of a large amount of images at different imaging modalities/scales: from atomic resolution for macromolecules (such as in protein crystallization), to subcellular locations (such as in location proteomics), up to human body organs or regions (such as in radiography).

In cell biology, the analysis of results of imaging experiments may provide biologists with new insights for a better understanding of all cellular components and behaviors [1]. However, visual classification (also called visual examination, phenotyping, recognition, categorization, labelling, sorting) of images into several classes with some shared characteristics (also called phenotypes, groups, types, categories, labels, etc.) is tedious. Indeed, manual classification of such an amount of images is time-consuming, repetitive, and is not always reliable, due to experimental conditions, variable image quality, and human subjectivity or tiredness that lead to considerable interobserver variations and misclassifications. In other words, manual examination could be a source of bias and would cause a bottleneck for high-throughput experiments, thus systems that automate image classification tasks would greatly help biologists. Ideally these systems should proceed faster than human in most cases, with the same accuracy (or even better when patterns are indistinguishable by human experts), and widely reduce the number of images that require human inspection (for example only in the case where the automatic system does not have a great confidence about its prediction).

In the computer vision community, image classification is a very active field. Given a set of training images labelled into a finite number of classes by an expert, the goal of an automatic image classification method is to build a model that will be able to predict accurately the class of new, unseen images. Such techniques have been applied to various problems where the goal is to identify a specific object (e.g. the face of a given individual, a particular building, someone's car), and current researches aim at developing generic methods for the categorization, detection and segmentation of classes of objects or scenes with shared characteristics in terms of their shapes, colors, and/or textures (cars, airplanes, horses, indoor/outdoor scenes, etc.) [2].

In the context of biomedical studies and cell biology, such automatic methods could for example help to study the phenotypic effects of drugs in human (red-blood) cells [3] where a class could denote the shape of a cell (stomatocyte, discocyte, or echinocyte). In various cytopathology studies, one may want to automatically recognize various cellular types to quantify their distributions in a certain state (e.g. cellular sorting in serous cytology [4]). Another promising example is the automatic identification of subcellular location patterns (e.g.: cytoplasm, mitochondria, nucleoli, etc.), using fluorescent tagging and fluorescence microscopy, as an essential first step to understand the function of various proteins [5,6]. Other recent examples of biological studies that can be formulated as image classification problems include the recognition of the different phases of the cell division cycle (interphase, prophase, metaphase, anaphase, etc.) by measuring nucleus shape and intensity changes in time-lapse microscopy image data [7,8], the microscopic analysis of urine particles (eg. squamous epithelial cells, white blood cells, red blood cells, etc.) [9], the study of protein distributions following a retinal detachment from confocal microscopy images [10], the annotation of fruitfly gene expression patterns over the entire course of embryogenesis obtained by *in situ *mRNA hybridization [11], etc.

### Related work

#### Global feature extraction

Till recently, image classification systems usually rely on a pre-processing step, specific to the particular problem and application domain, which aims at computing a certain number of numerical features from the initially huge number of pixels in images. Such features could for instance correspond to statistics of pixel intensities (mean, standard deviation, skewness, kurtosis, correlation between adjacent pixels, etc.), or compute various measures from preliminary segmented objects or "blobs" (ratio of area to perimeter, measure of straightness and curvature of boundaries, distance between objects, etc.), etc. This reduced set is then used as new input variables (also called features, signatures, descriptors) for traditional learning algorithms (for example a nearest neighbor or neural network classifier), possibly tuned for the specific application. The learning algorithm then tries to build from the data a model that associates features with predefined classes. The limitation of this approach is clear: a given set of features is suitable only for certain specific applications, but unsuitable for others, and the choice of which set of features to use for a given application is not obvious. Thus, when considering a new application or, more dramatically, when new image classes are of interest, it is often necessary to manually adapt the pre-processing step by taking into account the specific characteristics of the new task. Recently, several works tried to overcome this limitation and consider combining several different types of features that describe different aspects of an image, and applying feature selection techniques. In [5,7,12] several hundreds image features are extracted corresponding to texture descriptions, pixel intensity distributions, edges, responses to various filters, etc. However, these approaches that use global features may not work properly with cluttered and partially occluded images and they may not be robust to various image transformations (such as translation, orientation, scale, and viewpoint changes), that may appear in many applications. Meanwhile, it has been shown recently that generic methods developed by the object recognition community perform very well on medical images even though they were not tuned for such tasks [13].

#### Local appearance models

Many recent object recognition methods rely on a "local features" scheme [14-16]. First, interest points or image regions are detected (eg., by using a detector of peaks in local image variation) whose neighbourhood has high informational content and which are thought to be robustly detectable in images under varying conditions [17].

Then, the appearance of the interest points or regions is encoded by a feature vector of numerical values computed in their neighbourhood [18]. Such descriptors are often designed to be discriminative, concise and insensitive to various transformations that global feature methods are generally not able to cope with. These descriptors are sometimes compressed by dimensionality reduction techniques (such as Principal Component Analysis) because local regions contain too much data for the traditional learning methods that are not able to deal with very high numbers of variables. These local feature vectors are then stored in a database for use during the recognition step.

To predict the class of a new image, each feature vector computed from the image is classified using a nearest-neighbor algorithm against the feature vectors in the database. The majority class among the classes assigned to local feature vectors is then assigned to the image.

### Our work

In [19], we have proposed a generic approach for image classification that largely follows the aforementioned scheme but distinguishes from other methods by several notable points. First, the method uses a large set of *randomly *extracted image subwindows (or patches) and describes those by high-dimensional feature vectors composed by *raw pixel values*. Then, the method uses *ensemble of extremely randomized decision trees *[20] to build a subwindow classification model. To predict the class of a new image, the method aggregates subwindow class predictions given by the decision trees and it uses majority voting to assign a class to the image. Details about the method and its rationale are given in the Methods section.

Our approach was evaluated on various image classification datasets involving the classification of digits, faces, objects, buildings, photographs, etc. Moreover, in [21], we successfully applied it on a 10000 X-Ray image database with classification results very close to the best ones [13].

In this paper, we evaluate the potential of our image classification method in cell biology by evaluating its performances on four datasets of images related to protein distributions or subcellular locations and (red-blood) cells. The application of our method is straightforward (without incorporation of domain knowledge) and we compare its results with human classification (when available) and automated methods designed specifically for a given task. We discuss properties of the method such as attractive computational efficiency and possible interpretation.

## Results

The performance of our method is given for four image classification tasks: two of them correspond to sub-cellular protein localizations, the third one to red-blood cell shapes, and the last one to protein distributions in retina cells and layers. Details about these datasets are given in the Methods Section.

Basically we measure the accuracy of the models to correctly predict the class of unseen images. In all experiments, we build *T *= 10 trees using the default filtering parameter value (*k *= 256
 MathType@MTEF@5@5@+=feaafiart1ev1aaatCvAUfKttLearuWrP9MDH5MBPbIqV92AaeXatLxBI9gBaebbnrfifHhDYfgasaacH8akY=wiFfYdH8Gipec8Eeeu0xXdbba9frFj0=OqFfea0dXdd9vqai=hGuQ8kuc9pgc9s8qqaq=dirpe0xb9q8qiLsFr0=vr0=vr0dc8meaabaqaciaacaGaaeqabaqabeGadaaakeaadaGcaaqaaiabikdaYiabiwda1iabiAda2aWcbeaaaaa@2FAB@ = 16 for greyscale images, *k *= 768
 MathType@MTEF@5@5@+=feaafiart1ev1aaatCvAUfKttLearuWrP9MDH5MBPbIqV92AaeXatLxBI9gBaebbnrfifHhDYfgasaacH8akY=wiFfYdH8Gipec8Eeeu0xXdbba9frFj0=OqFfea0dXdd9vqai=hGuQ8kuc9pgc9s8qqaq=dirpe0xb9q8qiLsFr0=vr0=vr0dc8meaabaqaciaacaGaaeqabaqabeGadaaakeaadaGcaaqaaiabiEda3iabiAda2iabiIda4aWcbeaaaaa@2FBB@ = 28 for color images) except for the RBC task where we observed that its maximum value (*k *= 256) achieved better accuracy. The number of extracted subwindows is given for each problem. Details about our method and its parameters are given in the Methods Section.

### LifeDB

**Figure 1:**
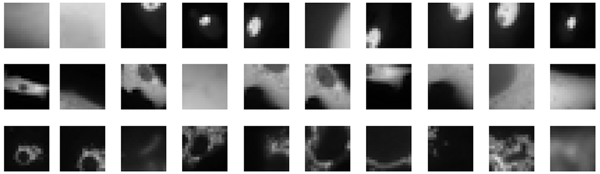
Examples of random subwindows extracted from images of the LifeDB dataset from classes nucleus (top), cytoplasm (middle), mitochondria (bottom).

Random guessing on this dataset would provide an error rate of 66.7%. Straightforward application of our method (with *N*_*ls *_= *N*_*test *_= 3000 subwindows extracted from each image) yields a leave-one-out prediction error equal to 6.45%. Examples of random subwindows extracted from these images are given in Figure 1.

Since for this experiment there are no results available from the literature, we applied a nearest neighbor classifier with euclidian distance and an Extra-Tree classifier on resized versions (200 × 100) of the global images (without subwindows extraction) to provide some baseline for comparison. With these methods, we obtained error rates of 33.33% and 11.82% (*T *= 500, *k *= 20000
 MathType@MTEF@5@5@+=feaafiart1ev1aaatCvAUfKttLearuWrP9MDH5MBPbIqV92AaeXatLxBI9gBaebbnrfifHhDYfgasaacH8akY=wiFfYdH8Gipec8Eeeu0xXdbba9frFj0=OqFfea0dXdd9vqai=hGuQ8kuc9pgc9s8qqaq=dirpe0xb9q8qiLsFr0=vr0=vr0dc8meaabaqaciaacaGaaeqabaqabeGadaaakeaadaGcaaqaaiabikdaYiabicdaWiabicdaWiabicdaWiabicdaWaWcbeaaaaa@3171@ = 141) respectively, which shows that the nearest neighbor classifier is here not able to deal with the high-dimensional feature vectors and the small number of images. On the other hand, the significant improvement of our method with respect to the Extra-Tree classifier confirms the interest of the subwindows sampling and voting scheme of our method.

### HeLa cells

Random guessing on this dataset would give about 90% error rate, while the human classification error rate on this task is of 17%, as reported in [22]. We obtain with our method an error rate of 16.63% ± 2.75 (when using *N*_*ls *_= *N*_*test *_= 2000).

We can compare these results with those of [23] (the first publication of this team based on this dataset) which range between 25% downto 15.6% depending on the number of features used and the parameters of the learning algorithm (a neural network classifier). Subsequently (see [12]), K. Huang and R.F. Murphy have improved these results downto 8.5% by using an unweighted majority-voting ensemble model of all possible combinations of eight classifiers, with several parameters optimized on this specific dataset.

**Figure 2:**
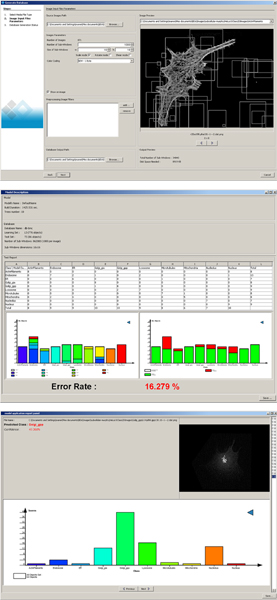
PiXiT screenshots when applied on HeLa cells. Parameter settings for the generation of learning set of subwindows (top); classification results of test images within one fold in terms of error rate, confusion matrix, confusion histograms (middle); prediction confidences for one image from class Golgi Gpp (bottom).

In terms of types of classification errors, let us notice that like the method presented in [22], our approach is more effective in distinguishing the two patterns of Golgi proteins (Giantin and gpp130) than human observers. On the other hand, errors of our approach are mostly due to misclassifications for the Endosome and Mitochondria classes. These results are further illustrated in Figure 2 which shows the confusion matrix of our method for one of the ten protocol executions (middle), as well as the prediction confidence for one Golgi Gpp image (bottom).

### Red blood cells (RBC)

In the literature, error rates on this dataset range from 31% to 13.5% [24], while the error rate of human experts is estimated to be above 20% [25]. On the other hand, with the protocol we used and due to the unbalanced number of images in each of the three classes, a method always guessing the most frequent class would achieve an 35.7% error rate. With our method, we obtained the best results by constraining the random subwindow sizes between 80% and 100% of the image size instead of the full range of sizes, with a mean error rate over all subsets of 20.92% ± 1.53 with 100 subwindows extracted from each image.

Notice that the method that obtains the best results on this dataset [24] also uses a local appearance approach, but with a distance measure between patches that incorporates invariances with respect to transformations that are known a priori: cell border line thickness, six affine transformations, and additive image brightness.

### Retinal detachment

In [10], authors proposed a method that computes different sets of MPEG-7 features within fixed-size square tiles, applies Independant Component Analysis to the feature vectors, and uses a Support Vector Machine classifier. Their results range from 65.6% downto 16.2% classification error rate on a dataset of 433 retinal images labelled into 9 classes. We obtain a 10% leave-one-out error rate using 5000 subwindows extracted from each image with subwindow random sizes inferior to 10% of the image size. Our 5 misclassification errors are confusions between "normal" and "1 day" conditions, and between "3 day" and "7 day" conditions. Our accuracy results are not directly comparable to those in [10] because the number of images and classes are not equivalent. However, they illustrate the ability of our method to capture the characteristics of these 4 classes using only a dozen images per class, hence its potential for this type of imaging experiments. A more in depth validation of our method on this type of problem would require a larger set of images representing additional experimental conditions (e.g. when different treatments are used).

**Figure 3:**
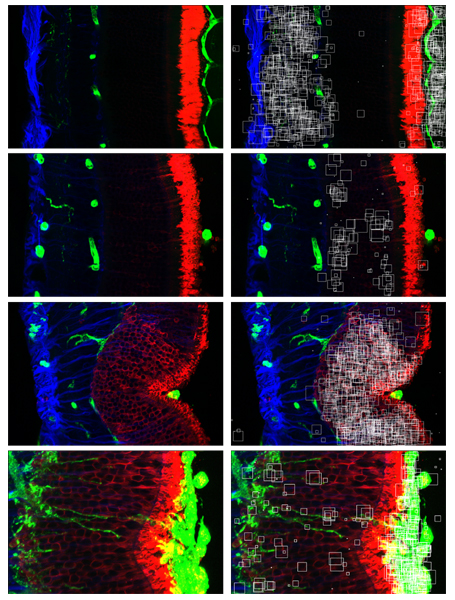
Discriminative subwindows on retinal detachment images. Left: one original image from each class (from top to bottom: normal, 1 day after detachment, 3 days, 7 days). Right: Discriminative subwindows among the 5000 randomly extracted subwindows per test image.

**Figure 4:**
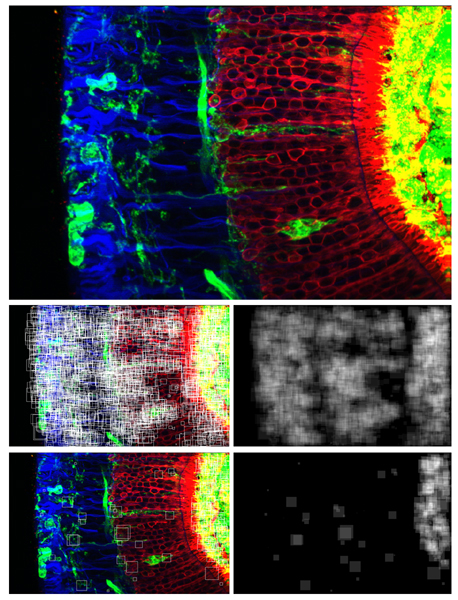
Subwindows and confidence maps on retinal detachment images. One original image from the class 7 day after detachment (top), 2656 correctly classified subwindows (among the 5000 randomly extracted) and confidence map (middle), 265 discriminative subwindows and confidence map (bottom).

Also, in order to be useful in practice, the image classification method should provide biologically meaningful information that can be interpreted by physicians, like for example the one used in [10]. As a first illustration of the possibility to gather such meaningful information with our method, Figure 3 shows the most discriminative subwindows of a particular image from each class, i.e. those subwindows that receive exactly *T *votes for that class (and no vote for any of the other three classes). Figure 4 shows for one image all the correctly classified subwindows and the most discriminative ones, with the corresponding confidence maps. The confidence maps are given in grey level images and show for each pixel the number of votes assigned to (correctly classified, or most discriminative) subwindows which contain the pixel. One can observe that the most discriminative regions of the image are identified by the confidence maps as those which indeed seem specific to the particular class. We believe that in specific studies, this kind of qualitative information could be quite useful for interpretation by domain experts.

## Discussion

We think our method is attractive for cell biology studies in view of its properties that we summarize hereafter.

First, without integrating any domain knowledge neither complex pre-processing techniques, our experiments show that our generic method obtains quite good results on average on four problems with images of different quality and representing various patterns. As one could have expected, these results are however not as good as the best results published in the literature obtained either with tailored methods for one specific dataset and/or after important research efforts (sometimes years of research).

Interestingly, our method is competitive with respect to classification by human experts on the HeLa cells and RBC tasks. In biological studies where the number of images to classify is so large, and where the perfect classification of molecules or cells is not required (but rather an estimation of distributions of types of cells, for example), the method would thus be quite useful. Indeed it is directly applicable to any image classification problem, it is reasonably fast, it can run on regular computers, and it would be easily possible to take advantage of parallel architectures, if available.

In the case of particular applications that require better prediction results than the ones obtained with the default settings of our method, its enhancement or tailoring is conceivable. Integration of domain knowledge would be possible. For example, in the case of protein subcellular localizations, the combination of the image classification and the classification of the amino acid sequence of the protein with a similar approach [26] might improve results. Domain knowledge could also be incorporated implicitly through the description of the subwindows with domain specific features, and also the exploitation of more generic image classification features (e.g. Haralick texture descriptors, Sobel edge features, etc.) may be useful. Generation of synthetic versions of the subwindows [27-30] might be another way to improve robustness (for e.g. to illumination changes or noise) by providing the learning method a richer training set to generalize from.

Beyond misclassification error rates, the method could highlight discriminative subwindows in images, hence it could be used as an exploratory tool for further biological interpretation. Preliminary results were given on the retinal dataset. For a specific study, this function should be applied on larger sets of images and corroborated by domain experts to assess its pratical usefulness.

## Conclusion

We illustrated the potential of our generic image classification method on different kinds of problems in cell biology. Thanks to its computational efficiency and competitive accuracy results on average with respect to human classification and tailored methods, we foresee the use of this automatic approach as a baseline method and a first try on various biological image classification problems where a manual approach could be a source of bias and would cause a bottleneck for high-throughput experiments. Moreover, preliminary results show that minor parameter tuning could possibly improve the default results on specific problems. Extension of this approach to image sequence classification and segmentation also deserves to be studied.

## Methods

We first describe the four image classification tasks and protocols used to evaluate our method. Our image classification method is explained afterwards.

### Image datasets

#### LifeDB

The subcellular localization of proteins is an essential step for the understanding of their function. The use of computer vision techniques for the recognition of patterns of subcellular fluorescence [31] is promising if combined with high throughput imaging systems [1,5,6]. In order to illustrate the potential of our method in that domain, we collected images from the website of an ongoing project about the localization of novel GFP-tagged human cDNA products to subcellular compartments of the eukaryotic cell [32,33].

**Figure 5:**
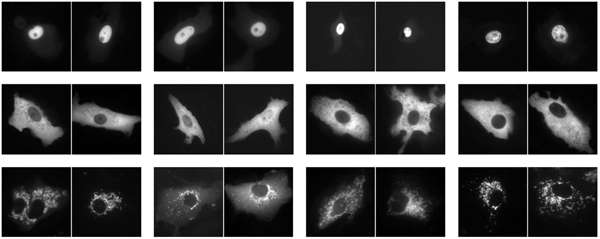
LifeDB. Pair of images for classes nucleus (top), cytoplasm (middle), mitochondria (bottom).

We selected 93 pairs of images corresponding to N- and C-terminal green fluorescent protein fusions of cDNAs [34] where the localization is visually identical whatever the fusion order is. The dataset thus contains pairs of greyscale images (2000 × 1000 pixels) of localized proteins into three intracellular compartments: nucleus (31), cytoplasm (31), and mitochondria (31), as illustrated in Figure 5.

As we collected the dataset by ourselves, we had to define a protocol to assess the classification performance. We used a leave-one-out error estimation as the dataset is rather small. That is, one model is built using all the images except one and the model is used to predict the class of the remaining image. The process is repeated for all the images, and the total number of prediction errors is counted. The total misclassification error rate is provided by percentage.

#### HeLa cells

**Figure 6:**
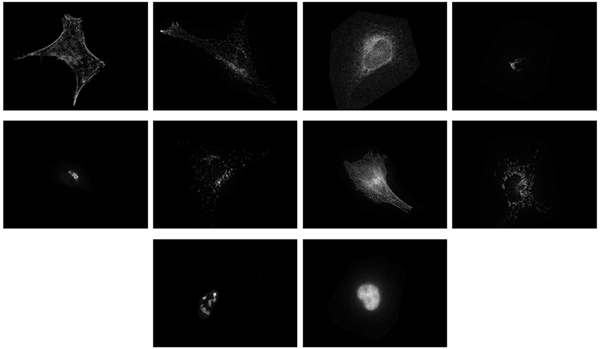
HeLa cells. From left to right, top to bottom: one image for each class actinfilaments, endosome, er, golgi gia, golgi gpp, lysosome, microtubules, mitochondria, nucleolus, nucleus.

Another experiment has been run for the localization of proteins on fluorescence microscope images in HeLa cells acquired by the Murphy Lab [35,36]. Images are labelled in ten different classes: ActinFilaments, Endosome, ER, Golgi gia, Golgi gpp, Lysosome, Microtubules, Mitochondria, Nucleolus, and Nucleus. This database contains 862 images of size 512 × 382 in greyscale, as illustrated by Figure 6. The number of images in one class varies from 73 (mitochondria) to 98 (actin filaments). We randomly picked 776 images for the training set (90% of 862) and tested the model on the remaining 86 images (10%). The procedure is repeated ten times, and the average error rate is provided.

#### Red blood cells (RBC)

**Figure 7:**
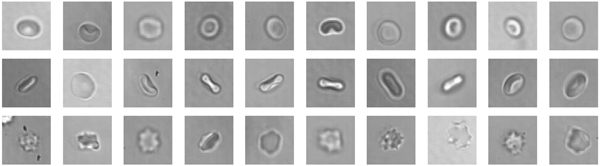
Red-blood cells. Images of classes stomatocytes (top), discocytes (middle), echinocytes (bottom).

Transitions in the shape of red blood cells (e.g. from the normal "discocyte" RBC toward echinocyte RBC) as the result of a drug is of particular interest in medical tests for drug discovery. However, visual inspection of shape changes of individual cells (per-cell classification) is a tedious manual labor. Thus a dataset [37] has been built to consider application of computer vision techniques in that field. The database contains 5062 RBC images that were labeled by an expert as either discocyte (916), stomatocyte (3259), or echinocyte (887). Each cell is represented by a 128 × 128 pixels sized grayscale image, as illustrated by Figure 7. The images were taken in a capillary where the RBC showed their native shapes without applied forces during sedimentation [38]. In addition to cell shape and intensity changes, images from a given class could appear with various transformations such as brightness variations, rotations in all possible angles and different cell border line thickness. This dataset was used previously by researchers at RWTH Aachen [3,25]. The dataset is split into 10 subsets (keeping the unbalanced class distribution), each subset is used for testing while the remaining 9 ones are used for training. The overall error rate is the mean over all subset error rates.

#### Retinal detachment

**Figure 8:**
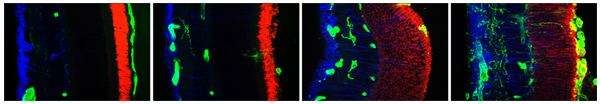
Retinal detachment. One image per class (normal, 1 day after detachment, 3 days, 7 days).

Examining patterns of distributions of proteins in cells to identify the differences and/or similarities between different stages of a biological process or disease is of particular interest for biologists. The retinal images from the UCSB Retinal Cell Laboratory and the Center for Bio-Image Informatics were collected to understand the structural and cellular changes of a retina following detachment. These color images represent the distributions of specific proteins in retinal cells and layers using antibody labelling followed by confocal microscope imaging. They were acquired in different experimental conditions that correspond to different stages of the retinal detachment process or that represent retinas exposed to different treatments [10]. We used the 50 publicly available color images [39] that represent 4 conditions illustrated by Figure 8: normal, 1 day after detachment, 3 days after detachment, and 7 days after detachment. Image sizes range from 630 × 420 to 1386 × 924 pixels. We used a leave-one-out protocol to evaluate classification accuracy.

### Random subwindows and extremely randomized decision trees

Given a set of training images labeled into a finite number of classes, the goal of an automatic image classification method is to build a model (training phase) that will be able to predict accurately the class of new, unseen images. The main characteristics of our method [19] are summarized as follows.

#### Training

During the training phase, a large number (*N*_*ls*_) of square subwindows of random sizes are extracted at random positions from each training image (see examples for LifeDB images in Figure 1). This random subwindow extraction provides a rich representation of images corresponding to various overlapping regions, both local and global, whatever the task and content of images. Each subwindow is then resized to a fixed size (16 × 16), to improve robustness to scale changes, and described by a high-dimensional feature vector of its raw pixel values (ie. 256 numerical values in the case of greyscale images, 768 in color images) to avoid discarding potentially useful information while being generic. Each subwindow is then labeled with the class of its parent image.

A subwindow classification model is then built by an ensemble of extremely randomized decision trees (Extra-Trees) algorithm [20]. This machine learning method has been shown effective (in terms of accuracy and computational efficiency) in a large variety of high-dimensional problems such as proteomic mass spectra classification [40] and DNA sequence classification [26]. Starting with the whole learning set of subwindows at the top-node, the Extra-Trees algorithm builds an ensemble of *T *fully-developed decision trees according to the classical top-down decision tree induction procedure [41]. The main difference between this algorithm and other tree methods is that while growing a tree, it splits nodes by choosing both attributes and cut-points at random. In the case of subwindow image classification, a binary test within a tree node simply compares the value of a pixel (intensity of a grey level or of a certain color component) at a fixed location within a subwindow to a cut-point value. In order to filter irrelevant attributes, the filtering parameter *k *corresponds to the number of attributes (ie. pixel locations) chosen at random at each node, where *k *can take all possible values from 1 to the number of attributes describing the subwindows. For each of these k attributes, a pixel intensity value threshold is randomly choosen. The score of each binary test is then computed on the current subwindow subset according to an information criterion [42], and the best test among the *k *tests is chosen to split the current node. The procedure is repeated recursively on subwindow subsets until the tree is fully developed. *T *fully-developed trees are built according to this scheme and saved (learning images and subwindows are no longer required for prediction).

#### Prediction

Classification of a new image similarly entails extraction and description of *N*_*test *_subwindows from this image, and the application of the model to these latter. Aggregation of subwindow predictions is then performed to classify the image, by assigning to the image the majority class among the classes assigned to each subwindow by each one of the *T *trees.

The method provides an interesting way to help domain experts to focus on discriminative regions in the images. Indeed subwindow individual votes are available when we predict the class of a new image. We can observe for each subwindow the distribution of votes for all classes assigned by the decision trees. The subwindows that receive the highest number of votes for a given class can then be considered as the most specific ones for that class and their visualization on the top of the image can bring potentially useful information about that class. Also, it is possible to generate a class-specific confidence map where each pixel corresponds to the sum of votes for that class received by every subwindows (correctly classified or only the most specific ones) the pixel belongs to. These functions are illustrated on the Retinal detachment images in the Results section.

#### Parameters and computational efficiency

The important parameters of the method are the number of subwindows extracted during learning (*N*_*ls*_) and prediction (*N*_*test*_), the number of trees *T*, and the extra-trees filtering parameter *k*. As a first try, we generally use a few hundred thousand of learning subwindows, a hundred or so subwindows per test image, and we build ten trees using the filtering parameter equal to the rounded square root of the number of attributes (default value suggested by [20]). As a general rule, we observe that the more subwindows we extract and trees we build, the better the accuracy is. Higher values of the filtering parameter also generally improve accuracy results. The parameter values could be adjusted in order to comply with desired computational efficiency requirements given that the complexity of the decision tree ensemble learning is on the order of *kT N*_*ls*_*logN*_*ls *_and that the prediction step is essentially proportional to *N*_*test*_*TlogN*_*ls*_. Note that the approach scales very well and, moreover, it is easy to parallelize.

#### Software

The above image classification method was implemented as a Java user-friendly software called PiXiT [43]. This software is freely available for research purpose. Screenshots of the software are shown in Figure 2. This software comes together with Annotor [44], a software developed by Vincent Botta which helps to annotate image databases. This second Java software allows users to annotate images through polygon labelling and to export individual annotations into directories of classes of images that can be imported into PiXiT to build classifiers.

## Competing interests

The authors have participated to the development of the PIXIT software package that is free to non-profit organisations but is commercially available for business use.

## Authors' contributions

The original image classification method was developed by RM, PG, and LW, with contributions of Justus Piater. The underlying machine learning method, extremely randomized trees, was developed by PG and LW, with contributions of Damien Ernst. RM carried out image classification experiments and drafted the manuscript, with revisions by PG and LW. All authors read and approved the final manuscript.
